# Sledge Hammer Impalement: a case report of challenges of managing a patient with a heavy pendant

**DOI:** 10.1016/j.ijscr.2020.05.026

**Published:** 2020-05-21

**Authors:** M.I. Ajaero, C.P. Echieh, C. Onyema, S. Nduagu

**Affiliations:** a Department of E.N.T., Federal Medical Centre Owerri, Nigeria; b Division of Cardiothoracic/Vascular Surgery, Department of Surgery, University of Calabar Teaching Hospital, Calabar, Nigeria

**Keywords:** Case report, Impalement injury, Zone 1

## Abstract

**Introduction:**

Impalement injuries are well defined. Transfixion injuries involve impalement and are defined in terms of fixation usually to a large object.

**Case Report:**

We report a spectacular case of sledge hammer impalement in the neck where the patient was transfixed, albeit to a small object, requiring movement of the patient and the transfixing object as a single unit.

**Discussion:**

The patient was fixed to the head of the sledge hammer because he was unable to move with the heavy pendant.

**Conclusion:**

We argue that transfixion injuries should be defined in terms of weight of the object in relation to the patient‘s weight and the ability of the patient to move (with) the impaling object.

## Highlights

• This patient suffered an impalement injury by falling on the upright handle of a sledge hammer.
• The hammer head was too heavy for patient transport hence patient was fixed to small sized but heavy hammer head.
• Patient had to be moved simultaneously with the sledge hammer.
• We argue that this meets the definition criteria for transfixion injury.
• We propose that transfixion injury be defined in terms of weight of the impaling object relative to the weight of the patient and ability to mobilize the patient and not necessarily by size of the impaling object.

## 1 Introduction

Impalement injuries are uncommon injuries [1], [2]. Even more uncommon are impalement injuries to the neck. Penetrating neck injuries can be associated with vascular, airway, cervical spine, or nerve injuries. The impaling foreign body may be providing tamponade if a major vascular structure is injured; hence, there is a possible benefit to leaving the foreign body in place until radiologic and surgical evaluations are performed [3].

In most cases, impalement injuries are caused by slender, lightweight objects. Rarely, the objects are long enough to compromise patient positioning and transport as a result of their sheer presence [1], [2], [4], [5], [6]. The aim of this case report is to discuss the challenges in the management of a patient who had impalement injury to Zone I region of the neck by a sledge hammer. This case report is unique because the weight of the impaling object, over 10% of the patient’s weight, was a heavy pendant which posed peculiar challenges to patient transport and positioning. The pendant had to be moved simultaneously with the patient to reduce the tangential torque it caused. This patient was managed at the tertiary medical centre by ENT and Cardiothoracic/Vascular surgeons. This case report is made in accordance with the S.C.A.R.E. guidelines [7].

## 2 Presentation of case

A 30-year-old male, manual worker who fell from the second floor of a construction site while demolishing a cast structure. The velocity of fall could not be estimated. He fell on a sledge hammer standing upright at ground level and presented with impalement injury to the neck. The Impaling object was a blunt ended wooden handle of a sledge hammer. The handle penetrated the left side of the neck with a trajectory in an anterior-posterior direction through the Zone I region of the neck with about 12 cm in the posterior aspect. The metal head was anterior and had to be supported to prevent dangling. Injury occurred in a remote community. He received initial resuscitation at a peripheral health facility close to the scene of the accident prior to referral. Necessary precautions during transport to referral centre were adopted by the transport team. Patient was transported to the referral centre in a private vehicle. During transport, he had episodes of fluctuating consciousness relieved by repositioning of the foreign body. Transit time to referral facility was 8 hours. He had no significant past medical history.

**Fig. 1:**
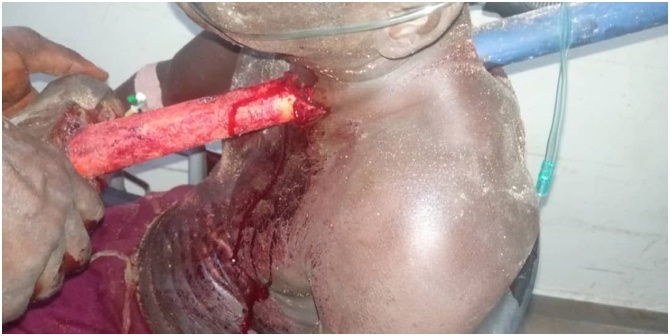
Showing the penetration of the neck by the foreign body.

**Fig. 2:**
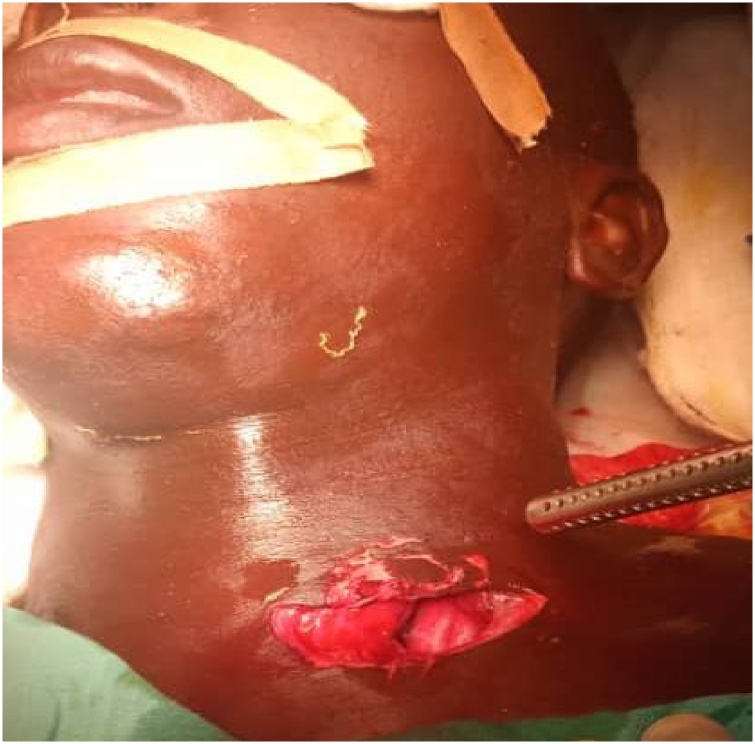
Showing neck wound after extraction of Foreign body.

On admission, physical examination showed a respiratory rate of 22 cycles per minute, pulse of 96 beats/minute, BP 140/80 mmHg. There was a neck wound with the foreign body protruding to the left of the midline just above the suprasternal notch (see Fig. 1). There was no subcutaneous emphysema around the neck. Examination of the abdomen, central nervous and musculoskeletal systems were unremarkable. Focused Assessment with Sonography for Trauma (FAST) as well as whole body trauma CT Scan were not readily accessible (Fig. 2).

Patient was wheeled into theatre with foreign body in situ, supported at the heavier end. Posterior limb of foreign body was carefully sawed at about 5 cm from the skin. Traction and counter traction were used to stabilize the foreign body while sawing. Patient was then laid supine and intubated with endotracheal tube. The neck was stabilized by placing the head on a ring and a sandbag between the scapulae. Routine skin preparation and draping was done. The shortened posterior limb of foreign body was disinfected and lubricated. The entry wound was extended to allow examination of vital structures as well as to determine the tract of the foreign body. The sledge hammer handle was seen passing medial to the carotid sheath and lateral to the oesophagus. No obvious injury was noted in the above two structures. The carotid sheath was not opened. The foreign body was extracted by gentle rotation and traction in posterior-anterior direction. Further neck exploration showed injuries to the platysma and the left sternocleidomastoid. The wound was subsequently debrided, irrigated and closed. Patient‘s weight after removal of foreign body was 77 kg. Weight of Sledge hammer and handle was 11 kg. Chest X-ray and neck CT Scan done in the post-operative period did not reveal any additional injury. Post-operative recovery was uneventful. Patient was discharged in the second post-operative week. Patient was followed up in the out–patient clinic for three months and was satisfied with the outcome of his treatment.

## 3 Discussion

The neck is vulnerable to whiplash injuries and penetration by sharp pointed objects. Impalement by blunt objects without associated injury to the crowded vital structures is unusual. This crowding mandate neck injuries be properly investigated and appropriately managed. The mortality from penetrating neck injuries is 2–10% and is related to haemorrhage from vascular injuries, respiratory and/or neurological problems [8]. We present a case of a 30-year-old manual worker who fell from height at a construction site. He had a sledge hammer impaled into zone I of the neck. The sledge hammer, though small in size, was too heavy for the patient to move around. Hence, the patient was essentially fixed and needed to be moved with hammer as a single unit.

Impalement injuries result from the penetration of fixed elongated objects through the body. They may present as overt or concealed injuries [9]. Type I impalement involves the decelerating human body falling on a stationary object, while a type II impalement involves a mobile object piercing through the stationary human body [1].

For purposes of penetrating neck injuries, the neck is divided into 3 zones. Zone I extends from the supra-sternal notch to the level of the cricoid cartilage. Some authorities consider the thoracic inlet as an inferior extension of the Zone I [8]. Zone II extends from the level of the cricoid cartilage to the angle of the mandible. Zone III extends from the angle of the mandible to the base of the skull.

Characterisation of internal injuries by means of X-ray, CT scan, MRI or FAST are necessary to determine treatment. However, the balance between detailed preoperative investigation and emergent operative intervention should be determined by physical examination findings and sound surgical judgement. It is reported that physical examination alone is safe and accurate for evaluation of vascular injuries in penetrating zone II neck injuries [10]. However, it is impossible to define all injuries from physical examination alone because aerodigestive tract injuries may not show on physical examination. The principle of intraoperative extraction of foreign body should be maintained because manipulation at the scene could relieve a tamponade on vascular injury leading to exsanguination. In our patient, careful precaution had to be taken during patient transport to prevent airway and/or carotid compression from the weight of the dangling head of the sledgehammer. It was surprising that despite the blunt end of the handle, this patient sustained a neat piercing injury through the neck without injury to nearby structures. We expected that the tensile strain resulting from driving the blunt end through the neck should have resulted in overpressure in the high-pressure carotid vessels. We also thought that the torque of the head at impact should have resulted in whiplash injury severe enough to cause unstable cervical spine injury. Typically, patients with impalement injuries present with an injury complex consisting of both blunt and penetrating injuries [1]. This patient did not have any identifiable blunt injury component.

During the surgery, we had anaesthetic challenges of positioning the patient for intubation. It was not possible to do endotracheal intubation in a lateral position as described by Udo et al because the head of the hammer was obstructing. We had to cut the posterior part of the wooden handle with a saw. This was to allow the patient to be positioned supine for endotracheal intubation and reduce the extraction distance of the foreign body. In this patient we opted for a cervical incision because there was no evidence of ‘hard signs’ of vascular injury. Contamination of such wounds is the rule, hence anti-infective measure such as wound care with debridement, irrigation and administration of antibiotics should be routine. Outcome of such injuries depend on extent of injury, and involvement of vital structures.

Impalement injury is a rare type of mechanical injury following forceful insertion of a projecting object into the body [5]. In contrast, transfixion injury is a type of penetrating injury in which the body is pinned onto a fixed object [11]. In such cases, movement of the patient would require simultaneous movement of the transfixing object and the patient as a single unit. Extrication of the patient may require a disassembly of the transfixing object. In our patient, the sledge hammer handle pinned the patient to the sledge hammer head. As a result, movement of the patient could lead to a shearing force if the hammer and patient were not moved as a unit. We argue that our case met the criteria for transfixion injury and that size of the object should not be a factor in the definition of transfixion injury. Case definition of transfixion injury should be based on weight of the object and the ability of the patient to drag the object around. We recommend weight of impaling object more than 10% of body weight and patient‘s inability to ambulate with the impaling object.

Another unique feature of this case is the external compression of the carotid vessels resulting in cerebral ischaemia and fluctuating consciousness. We are convinced that the external compression was from the weight of the hammer head transmitted via the handle and not just from the presence of the handle in the compact neck.

## 4 Conclusion

This case was unique case in which the patient was transfixed to a small sledge hammer head.

## Conflict of interest

No conflict of interest declared.

## Funding

No funding was obtained for this study.

## Ethical approval

Ethical approval was waived.

## Consent

Permission of the patient to publish this case was obtained.

## Author contribution

M. I. Ajaero: lead surgeon, approved the final manuscript.

C. P. Echieh: attending surgeon, conceptualization, prepared the manuscript.

C. Onyema: attending surgeon, conceptualization, reviewed the manuscript.

S. Nduagu: attending surgeon, reviewed the manuscript.

## Registration of research studies

1. Name of the registry: Not applicable.
2. Unique identifying number or registration ID: Not applicable.
3. Hyperlink to your specific registration (must be publicly accessible and will be checked): Not applicable.

## Guarantor

M. Ajaero.

## Provenance and peer review

Not commissioned, externally peer-reviewed.
